# Use of a cervical stent for long‐term treatment of pyometra in the mare: A report of three cases

**DOI:** 10.1111/rda.13480

**Published:** 2019-06-26

**Authors:** Judith Krohn, Sophia Ennen, Rainer Hospes, Jennifer Nieth, Axel Wehrend

**Affiliations:** ^1^ Department of Veterinary Sciences, Clinic for Obstetrics, Gynecology and Andrology of Large and Small Animals Justus‐Liebig‐University Giessen Germany; ^2^ Veterinary Group Practice Rodewald Rodewald Germany

**Keywords:** cervix, mare, permanent catheter, pyometra

## Abstract

An effective long‐term treatment is necessary for mares with pyometra, because the condition tends to recur. In many affected animals, several conformational or anatomical anomalies contribute to impaired uterine clearance. Ovariohysterectomy is the surgical procedure of choice. Conservative therapy consists of draining and flushing the uterus, and systemic anti‐inflammatory and antimicrobial treatment. Uterine secretions tend to accumulate again after local treatment, especially in mares with poor vaginal conformation or cervical adhesions. Herein, we describe three cases in which a cervical stent was used in mares after mechanical or manual dilation of the cervix to achieve permanent draining of the uterus. The mares remained symptom‐free for up to 6 years and exhibited good clinical progress and good performance in competitions. Potential complications of the procedure include loss of the stent and obstruction caused by viscous secretion. A cervical stent is a relatively easy and low‐cost option for the long‐term treatment of pyometra in mares, particularly in cases where excessive costs of surgery and risks of a general anaesthesia are to be avoided.

## 1 INTRODUCTION

The accumulation of large amounts of inflammatory fluid in the uterine cavity of mares is referred to as pyometra (Santschi et al., 1995). The inflamed area can be sterile or it can harbour bacteria. The causes are a combination of poor vaginal conformation (Hurtgen, 2006), impaired cervical function and reduced uterine clearance. Pyometra can be considered a chronic inflammatory condition that develops from persistent clinical endometritis. This is an important risk factor for subfertility or even infertility in mares (Hurtgen, 2006; LeBlanc & Causey, 2009).

Reduced uterine clearance is multifactorial process, but cervical damage or malfunction is the most important reason resulting in pyometra. Hereditary malformations of the cervix (Wehrend, Herfen, Litzke, & Bostedt, 2000) or the caudal reproductive tract can result in pyometra (Egloff et al., 2013). Cervical laceration during dystocia or mating can lead to the formation of scar tissue, adhesions and fibrosis, which can reduce mechanical elimination of pathogens (Sertich, 2007). Impaired cervical closure is associated with a higher risk of bacterial contamination of the uterus. The passage of bacteria and foreign substances is normally prevented by tight closure and thick mucus in the cervical canal (Brinsko et al., 2010).

In mares, pyometra can occur spontaneously or after insemination. In some cases, clinical symptoms are completely absent, and where present, they are highly variable and can include non‐specific weight loss, reduced performance, mild fever, colic or combinations thereof (Pycock, 2007). In a transrectal examination, the uterus presents enlarged and fluctuating, and ultrasound reveals echogenic fluid (Brinsko et al., 2010). If the cervix is not completely closed, vaginal discharge may be observed. Sometimes pyometra is incidentally diagnosed during routine gynaecological examination. In a vaginal examination, spontaneous discharge may be present. In cases involving cervical adhesions, mares often do not exhibit discharge unless the cervix is opened manually (Fedtke, Traeneckner, & Kreling, 2010).

The standard treatment for pyometra consists of mechanical draining of the exudate and flushing of the uterus and enhancement of uterine contractions via oxytocin. If symptoms of systemic inflammation are present, a systemic antimicrobial treatment can be effective. Complete recovery can be achieved (Shamar & Tapak, 2010) but is unlikely. In many cases, mares can recover from an acute state, but they remain at high risk of recrudescence. Ovariohysterectomy is a surgical treatment option (Rötting, Freeman, Doyle, Lock, & Sauberli, 2004). Chronic inflammation and potential contamination of the uterus increase the risks of postoperative complications above those routinely associated with general anaesthesia in horses.

Permanent drainage of the uterine lumen is essential, especially if cervical adhesions prevent the efflux of fluid. This can be achieved via a Foley catheter, which can be used for several days or weeks (Neuhauser & Handler, 2008). Notably however, this is not a viable long‐term option. A permanent stent that allows continuous drainage of the endometrial secretion is preferable.

## 2 CASE PRESENTATIONS

All cases were presented to the Clinic of Gynecology, Andrology and Obstetrics of the Justus‐Liebig University of Giessen between 2010 and 2017. All experimental procedures were approved by the Ethics Committee of Regierungspraesidium Giessen, Germany (V 54‐19c20 15h02Gl18/14kTV13/2017).

### 2.1 Case 1

**Figure 1:**
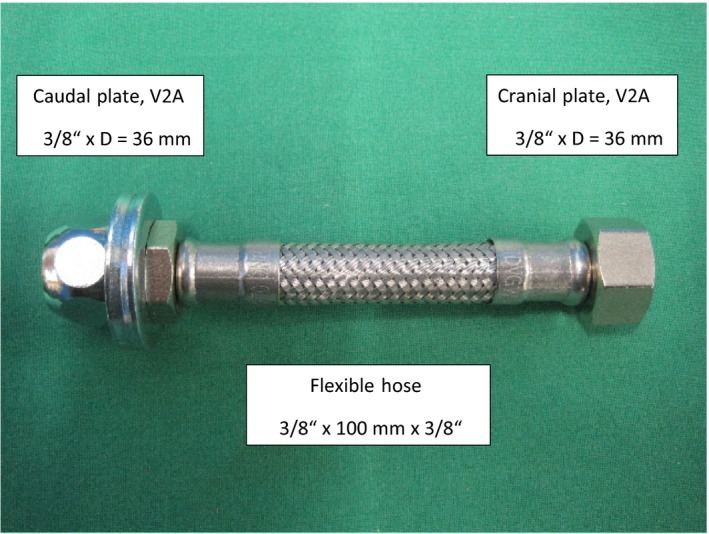
Permanent catheter placed in the cervix for drainage of the uterine lumen

A 19‐year‐old paint horse mare was presented in October 2010 with fever and colic symptoms, and gynaecological examination revealed pyometra. The mare had given birth to 1 foal several years ago without complications. The vagina and cervix exhibited massive adhesions. Manual passage of the cervical canal was possible, and the uterus was flushed with saline. The mare was treated with antibiotics and non‐steroidal anti‐inflammatory drugs for 7 days. Over the next 18 months, the mare regularly exhibited symptoms of colic and fever, and there was constant accumulation of fluid in the uterine lumen. In February 2012, a permanent catheter (stent) was placed in the cervix under sedation and epidural anaesthesia. Beforehand, the cervical canal was dilated with a balloon used for catheter interventions in small animal surgery. The stent was manufactured by G. Herber, a technician of the faculty of veterinary medicine according to the authors' requirements. It consisted of a flexible connecting hose (length 10 cm) used for sanitary installations combined with a plate and screw nuts at both ends (Figure 1).

**Figure 2:**
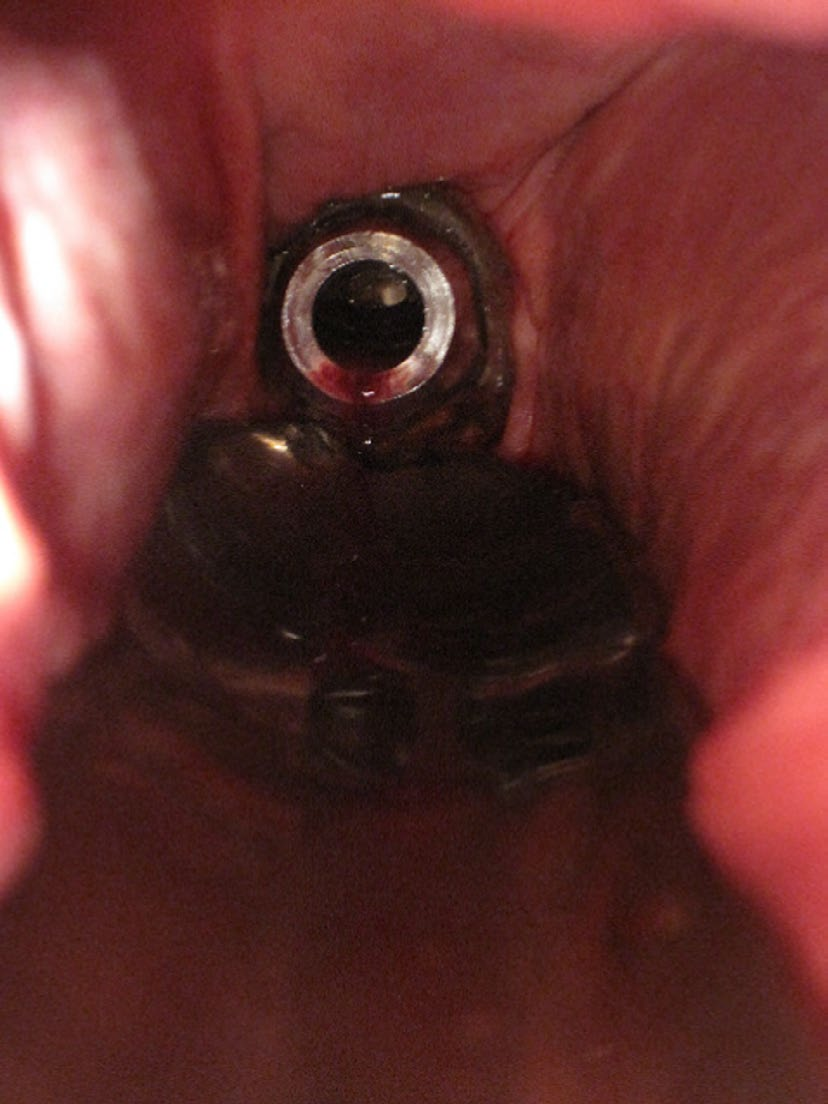
Vaginoscopic picture of the stent in the cervix

The mare was discharged 2 days later and examined at regular intervals. The stent was in place at all times (Figure 2). There was sufficient drainage from the uterine lumen, there were no signs of colic or pain, and transrectal sonography indicated that there was only mild accumulation of fluid in the uterus (1–2 cm at the point of largest diameter). She exhibited good body condition and no general signs of illness 2 years later.

Six years after the intervention, the owners noted that the mare was showing signs of discomfort during urination. A few days later, they found the cervical stent in the stable. Three weeks later, the mare was presented for examination at the clinic. In the general examination, she did not show abnormalities. Gynaecological examination revealed the accumulation of 3 cm of echogenic fluid at the point of largest diameter in the uterine lumen. Vaginal examination revealed massive adhesions in the vaginal canal, and the cervix could not be visualized or palpated. As the accumulation of fluid was only minor, the owner decided to re‐evaluate clinical progress at a later date rather than instigating another catheter intervention. Re‐evaluation was performed 8 months later: the mare had shown regular vaginal discharge, and filling of the uterus did not exceed the volume discovered during the previous examination. The vagina still showed massive adhesions but the rudimental cervical canal seemed to allow passage of secretion.

### 2.2 Case 2

An 18‐year‐old warmblood mare was presented with colic and fever 8 weeks after her last insemination. She had given birth to one foal several years previously, had failed to become pregnant earlier in that season, but was diagnosed as being pregnant with large amounts of foetal fluid 2 weeks before presentation. To support luteal function, she had been administered the gestagen altrenogest over the last 4 weeks. Due to the colic, the mare had been treated with anti‐inflammatories and antibiotics, without success. During a gynaecological examination, 35 L of purulent secretion was removed from the uterus. Passage through the cervix was easy, but palpation revealed a solid consistency presumably due to fibrosis. Flushing of the uterus, oxytocin injections and antimicrobial therapy were carried out. Bacteriological culture from the uterus revealed moderate amounts of *Klebsiella pneumoniae* and *Streptococcus equi* subspecies *zooepidemicus*. The measurement of equine chorionic gonadotropin (2 ng/ml) indicated that she had not been pregnant for more than 35 days.

**Figure 3:**
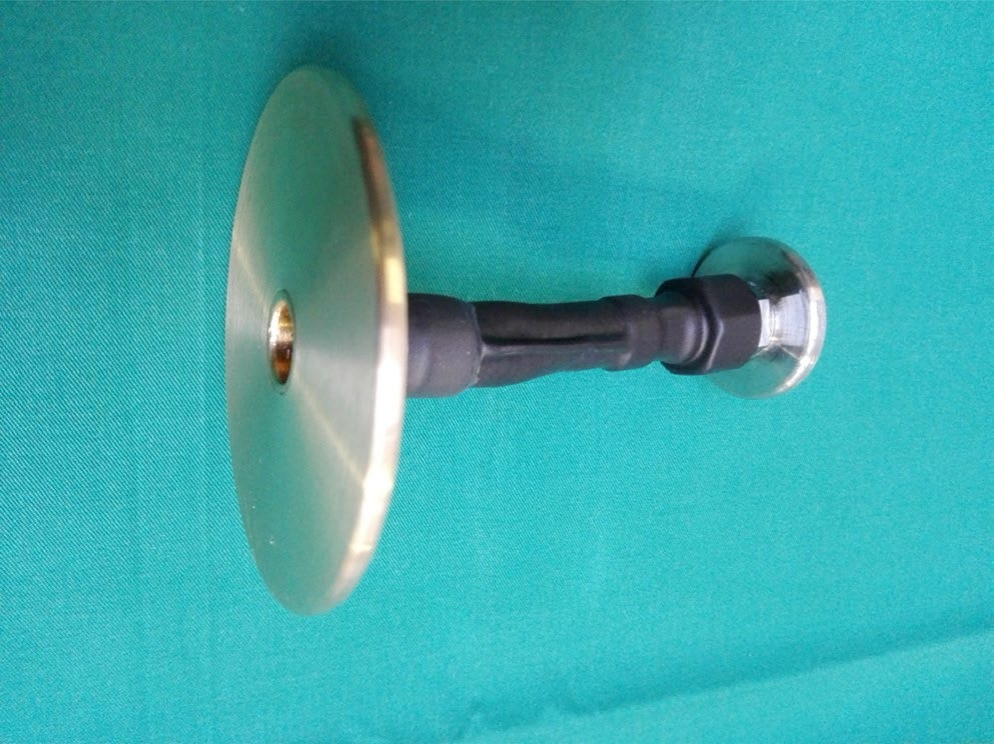
Cervical stent with enlarged endpiece (8 cm in diameter) covered by a shrinking tube for implantation into a dilated cervix

As the mare exhibited recrudescent filling of the uterus with purulent secretion during the following weeks, a cervical stent was administered 3 weeks later. To avoid corrosion, the stent was covered by a shrinking tube, as a modification to the stent used in case 1. The catheter had to be replaced with a system with a larger caudal plate (8 cm in diameter; Figure 3) because it slipped into the uterine lumen 2 days after implantation. The stent has remained in the cervical canal since this modification.

The mare was presented for regular control examinations between 2014 and 2018. She exhibits intermittent vaginal discharge, but is generally in good condition. She is successfully performing in high‐class jumping competitions.

### 2.3 Case 3

A 15‐year‐old appaloosa mare was presented in August 2017 with vaginal discharge. She had been inseminated several times 3 years prior, but had never conceived. During the cycle before presentation, the owner noted vaginal discharge. The mare showed regular oestrous signs. Transrectal examination revealed that the uterus was filled with purulent fluid.

Bacterial culture revealed a large amount of *S. zooepidemicus* and a small amount of *K. pneumoniae*. The mare was treated with daily flushing of the uterus and oxytocin injections. After 5 days, less than 3 cm of clear fluid was discovered in the uterine horn at the point of largest diameter. In an examination 3 weeks later, the findings were identical. A further 8 weeks later, the uterus was filled with 1 L of fluid. A stent was inserted into the cervical canal under sedation. The stent had since remained in place for 7 months, and the amounts of uterine fluid detected during intermittent examinations did not exceed 1 cm at the point of largest diameter. The mare was in good clinical condition and performing well during exercise. Nine months after implantation, the mare was examined due to increasing amounts of vaginal discharge. The stent had moved 2 cm cranially into the fibrotic cervix. Under sedation and epidural anaesthesia, the cervix was manually dilated, and the position of the stent was corrected. The owner decided she wanted the mare to be ovariohysterectomized, so the stent was removed. Surgery was performed uneventfully. Postoperative complications consisted of a wound infection and later on recrudescent filling of the uterine stump.

## 3 DISCUSSION

Pyometra is relatively rare in mares compared with other species, such as dogs (Egenvall et al., 2001). In affected animals, the prognosis for reestablishment of breeding capacity is poor. Permanent closure of the cervix leads to a closed form of pyometra with large amounts of intrauterine fluid (Fedtke et al., 2010).

Therapeutic options include surgical and non‐surgical procedures. The most common surgical option is complete resection of the ovaries and uterus (ovariohysterectomy). Resection of the enlarged and fluid‐filled uterus can be difficult, and the risk of peritonitis or wound infection is increased due to potential contamination of the abdomen. Furthermore, the complete removal of the uterus is impossible and remaining uterine tissue puts the mare at a high risk to develop postoperative complications. Thus, this surgery is a less common treatment in horses than it is in other species such as dogs (Bloomberg, 1996). An advantage of a laparotomy is the possibility of detecting abdominal adhesions or other abnormalities as a cause of recurrent colic (Butson, England, & Blackmore, 1996). Laparoscopic dissection of the mesovarium and mesometrium has facilitated ovariohysterectomy in the mare (Woodford, Payne, & Mc Cluskie, 2014). Another surgical option is the cervical wedge resection described by Arnold et al., who achieved permanent drainage of the uterus in mares with intraluminal cervical adhesions (Arnold, Brinsko, & Varner, 2015). Especially in mares with very pronounced cervical adhesions as described in case 1, the wedge technique can be difficult to perform and has a questionable success.

A non‐surgical option to treat pyometra is draining and flushing of the uterus. To extend the period of drainage, a Foley catheter can be placed into the cervix for several days to allow complete emptying of the uterine lumen (Neuhauser & Handler, 2008). In many cases, mares can exhibit recrudescence, especially if uterine irritation (e.g., via mating or insemination) occurs. In some cases, flushing can eliminate symptoms for months or years (Shamar & Tapak, 2010). If this therapy is not effective, permanent draining of the uterus is recommended. In the above‐described cases, permanent draining in recrudescent mares was achieved using a permanent cervical stent. To avoid corrosion of the material and smooth the surface, a shrinking tube was administered in cases 2 and 3. A shrinking tube is a rubber tube that can be placed over pipes or cables and thermally shrinks to insulate connections. Reactive inflammation due to mechanical irritation is a common complication. This was not a major problem in any of the above‐described cases. No discomfort was reported by the owners during rest or exercise, following the procedures. By utilizing the stent intervention detailed above, a surgical procedure and the associated costs and risks may be avoided, and thus, it constitutes an advantageous alternative for both the mare and its owner. Moreover, implantation of the stent in most mares is possible with minimal effort and therefore a convenient option for practitioners.

In our opinion, the cervical stent is a cost‐effective, minimally invasive and conservative treatment that is appropriate for use in mares—particularly in cases where general anaesthesia should be avoided for reasons such as age, systemic disease or costs. A good clinical condition can be achieved via the procedure. Regular transrectal and transvaginal examinations are advisable to confirm that the stent remains in place and functional. In mares with heavy cervical or vaginal adhesions (as in case 1), stent implantation can be difficult, but in the majority of mares, manual dilation of the cervix under mild sedation, and if necessary epidural anaesthesia, is adequate.

Adverse side effects and risks associated with the procedure are marginal. Loss of function can occur if the stent is lost, shifts in the cervical canal or is blocked by detritus. If necessary, the lumen can be cleared by careful flushing with saline at regular intervals to dilute viscous secretions. In the follow‐up examinations of the above‐described cases, none of the mares exhibited blockage of the stent, but they had exhibited regular vaginal discharge. Loss of the first stent occurred in case 2 very soon after implantation because that stent did not fit tightly enough into the mare's dilated cervical canal. Adjustment of the pieces of the stent resulted in a secure fit in the cervix. A larger piece at the caudal part prevents movement into the uterine lumen. The stent was lost several years after implantation in case 1, and before this occurred, it had not been re‐evaluated for several years. Otherwise, the displacement of the catheter could have been detected and corrected earlier. Nevertheless, the asymptomatic interval was 6 years. The persistency of vaginal discharge after loss of the stent suggests that during the long time in place, the stent leads to structural changes in the fibrotic cervix which now prevents complete closure.

In conclusion, pyometra in mares is a devastating disease in terms of breeding capacity but adequate treatment can result in a good condition without clinical symptoms and good general performance.

## CONFLICT OF INTEREST

None of the authors have any conflict of interest to declare.

## AUTHOR CONTRIBUTIONS

J. Krohn: examined and treated all mares, developed the modification of the stent and wrote and revised the manuscript. S. Ennen: investigated the mare presented in case one and was involved into in invention and implantation of the first stent. She revised the manuscript. R. Hospes: was involved into invention and implantation of the first stent and folllow up examinations in case one. He revised the manuscript. J. Nieth: examined and treated the mare presented in case three. She was involved into implantation and modification of the stent in case three and revised the manuscript. A. Wehrend: supervised examination and treatment of all presented cases and revised the manuscript.

## Data Availability

The data that support the findings of this study are available from the corresponding author upon reasonable request.
